# Carotid Intima-media Thickness in Patients with Non-alcoholic Fatty Liver Disease Attending a Tertiary Care Center: A Descriptive Cross-sectional Study

**DOI:** 10.31729/jnma.5179

**Published:** 2021-05-31

**Authors:** Bom BC, Raj Kishwor Jaiswal, Prashant Kumar Gupta, Rajan Paudel, Raj Kumar Subedi

**Affiliations:** 1 Department of Radiology, Rapti Academy of Health Sciences, Dang, Nepal; 2 Department of Radiology, Bir Hospital, Kathmandu, Nepal; 3 Central Department of Public Health, Institute of Medicine, Tribhuwan University, Kathmandu, Nepal; 4 Bhaskar-Tejshree Memorial Foundation, Kathmandu, Nepal

**Keywords:** *body mass index*, *fatty liver disease*, *ultrasound*

## Abstract

**Introduction:**

Non-alcoholic fatty liver disease is fatty infiltration of the liver in the absence of other causes of steatosis. It is strongly associated with central adiposity, high body mass index, insulin resistance states, hypertension, hyperlipidemia which are features of metabolic syndrome. The objective of study is to find out the carotid intima-media thickness of non alcoholic fatty liver disease patients attending a tertiary care center.

**Methods:**

This was a descriptive cross sectional study conducted at National Academy of Medical Sciences, Bir Hospital from July 2018 to June 2019 among 70 diagnosed cases of non alcoholic fatty liver disease based on ultrasound findings. Data collection was started after receiving ethical approval from Institutional Review Board of the Institute. Convenience sampling method was used. Data were entered using Microsoft Excel. The carotid intima-media thickness of both sides were measured by ultrasound. Statistical Package for Social Sciences version 20 was used for analysis.

**Results:**

Out of 70 cases, the mean carotid intima-media thickness was 0.7140±0.1796mm on right and 0.7161±0.1828mm on left side. Among 70 cases 45 (64.3%) were Grade II non alcoholic fatty liver disease and 25 (35.7%) were Grade I. It was 0.5720±0.1275mm and 0.7929±0.1546mm in Grade I and II non alcoholic fatty liver disease cases respectively on right side whereas it was 0.5676±0.1259mm and 0.7987±0.1557mm respectively on left side.

**Conclusions:**

This study showed increased carotid intima-media thickness in non alcoholic fatty liver disease cases.

## INTRODUCTION

Non-alcoholic fatty liver disease (NAFLD) is fatty infiltration of the liver in the absence of other causes of steatosis, such as alcohol consumption characterized by excessive fat accumulation in liver >5-10% of its weight encompassing a spectrum of increasingly severe clinico-pathological conditions - nonalcoholic fatty liver (NAFL) and nonalcoholic steato-hepatitis (NASH) with or without fibrosis/cirrhosis.^1-3^

The epidemic proportions of NAFLD prevalence is due to sedentary lifestyle, metabolic syndrome and obesity which is correlated to body mass index (BMI), fat distribution, race, ethnicity and sex with several studies showed the association between hepatic steatosis and carotid atherosclerosis.^2,4,5^ The overall sensitivity, specificity, positive likelihood ratio, and negative likelihood ratio of ultrasound for the detection of moderate-severe fatty liver were good compared to histology (gold standard) in different studies.^6,7^

So, in this study, we attempt to find carotid intima-media thickness of non alcoholic fatty liver disease patients in different grades of fatty liver, body mass index, place of residence and ethnicity.

## METHODS

This was a hospital based descriptive cross-sectional study carried out at National Academy of Medical Sciences (NAMS), Bir Hospital Kathmandu from July 2018 to June 2019. Data collection was started after obtaining an approval from the Institutional Review Board of NAMS (Reference no. 223 dated on 12th Asadh 2075) and informed written consent was also taken from each patient. Patients between 30 to 60 years of age with ultrasonic findings of hyper-echoic liver with negative history of alcohol abuse, either total abstainers or who consumed <20g of alcohol per day (The diagnosis of NAFLD requires the exclusion of alcohol abuse as the cause of liver disease; a daily intake as low as 20g in females and 30g in males may be sufficient to cause alcohol-induced liver disease in some patients (350ml [12 oz] of beer, 120ml [4 oz] of wine, and 45ml [1.5 oz] of hard liquor each contain 10g of alcohol)^8,9^ were labeled as NAFLD with other causes, such as viruses, autoimmune responses, metabolic or hereditary factors, and drugs or toxins were ruled out. Patients with clinical evidence of chronic liver conditions (e.g. known case of liver cirrhosis, viral hepatitis, autoimmune hepatitis, use of hepato-toxic drugs), renal disease, cardiovascular events, recent history of acute illness, age >60 yrs or <30 yrs or alcohol consumption >20g/day were excluded from this study.

The sample size was calculated using the following formula,


$$\begin{array}{ll} \text{n} & \text{= }{\text{Z}}^{\text{2}}\times {\text{σ}}^{\text{2}}/{\text{e}}^{\text{2}}\\ & \text{= }{\left(1.96\right)}^{\text{2}}\times {\left(0.18\right)}^{2}/{(\text{0.05)}}^{\text{2}}\\ & \text{= }50 \end{array}$$

Where,

n = minimum required sample size,
Z = 1.96 at 95% Confidence Interval (CI)
σ = standard deviation taken from a previous study, 0.18^10^
e = margin of error, 0.05

Total sample size was 50. But we took 70 cases for our study. Convenience sampling was used to collect the data.

Data obtained were entered into the computer using Microsoft Excel programs with their statistical analysis and relevant statistical tests along with generation of tables using Statistical Package for Social Sciences (SPSS) version 20 software.

## RESULTS

Out of 70 cases, the mean carotid intima-media thickness was 0.7140±0.1796mm on right and 0.7161±0.1828mm on left side. Among 70 cases, 45 (64.3%) were Grade II non alcoholic fatty liver disease (NAFLD) and 25 (35.7%) were Grade I while there were no Grade III NAFLD cases. Maximum NAFLD cases of 16 (22.9%) were seen in 30-35 years age group followed by 46-50 and 51-55 years age groups. About 36 (51.4%) were female and 34 (48.6%) were male, out of these 70 cases. Thirty seven (52.9%) were Brahmin/Chhetri as maximum participants followed by Janajati, Madeshi and Dalit. Forty five (64.3%) participants were from outside the Kathmandu valley while 25 (35.7%) were from Kathmandu valley. Forty one (58.6%) participants were overweight, while 19 (27.1%) were obese and 10 (14.3%) were normal with respect to their BMI (Table 1).

**Table 1:** Socio-demographic characteristics of non-alcoholic fatty liver disease (NAFLD) patient.

Characteristics	NFALD Grading		Total n (%)
	I n (%)	II n (%)
**Age (years)**			
30-35	9 (36)	7 (15.6)	16 (22.9)
36-40	4 (16)	8 (17.8)	12 (17.1)
41-45	1 (4)	6 (13.3)	7 (10)
46-50	8 (32)	7 (15.6)	15 (21.4)
51-55	2 (8)	11 (24.4)	13 (18.6)
56-60	1 (4)	6 (13.3)	7 (10)
**Sex**			
Female	13 (52)	23 (51.1)	36 (51.4)
Male	12 (48)	22 (48.9)	34 (48.6)
**Ethnicity**			
Brahmin/Chhetri	16 (64)	21 (46.7)	37 (52.9)
Dalit	2 (8)	3 (6.7)	5 (7.1)
Janajati	5 (20)	17 (37.8)	22 (31.4)
Madhesi	2 (8)	4 (8.9)	6 (8.6)
**Place of residence**			
Inside Kathmandu valley	9 (36)	16 (35.6)	25 (35.7)
Outside Kathmandu valley	16 (64)	29 (64.4)	45 (64.3)
**Body weight**			
Normal	5 (20)	5 (11.1)	10 (14.3)
Overweight	16 (64)	25 (55.6)	41 (58.6)
Obese	4 (16)	15 (33.3)	19 (27.1)

The overall minimum & maximum CIMT was 0.36mm and 1.2mm respectively. Similarly minimum & maximum CIMT were 0.36mm & 0.81mm respectively for Grade I NAFLD and 0.45mm & 1.20mm respectively in Grade II NAFLD on right side while it was 0.36mm & 0.81mm for Grade I NAFLD respectively and 0.49mm & 1.20mm respectively in Grade II NAFLD. The overall mean CIMT was 0.7151±0.1805mm. Mean CIMT was 0.7140±0.1796mm on right side while 0.5720±0.1275mm and 0.7929±0.1546mm in Grade I and Grade II NAFLD respectively. Similarly, it was 0.7161 ±0.1828mm on left side with while 0.5676±0.1259mm and 0.7987±0.1557mm in Grade I and II NAFLD respectively. The mean CIMT values of both sides were higher in grade II as compared to grade I NAFLD suggesting higher values are observed in higher grades of fatty liver (Table 2).

**Table 2:** Carotid intima-media thickness (CIMT) and non-alcoholic fatty liver disease (NAFLD) grading.

CIMT	NAFLD Grade	No.	Mean	Std. Deviation	95% Confidence Interval of	
					Lower	Upper
CIMT Rt.	I	25	0.572	0.12748	-0.29336	-0.14842
(mm)	II	45	0.7929	0.15459		
CIMT Lt.	I	25	0.5676	0.12594	-0.30367	-0.15846
(mm)	II	45	0.7987	0.15566		

Studying the observed mean differences as per BMI showed increased CIMT values in both sides in obese (BMI≥30kg/m^2^) as compared to normal (BMI<25) and overweight (BMI≥25kg/m^2^ and <30kg/m^2^) cases. Likewise, there were increased CIMT values in overweight as compared to normal BMI cases. These results suggest that CIMT values are increased in high BMI cases (Table 3).

**Table 3:** Carotid intima-media thickness (CIMT) as per BMI in NFALD patient.

Dependent Variable	(I) BMI	(J) BMI	Mean	95% Confidence Interval	
	recoded	recoded	Difference (I-J)	Lower Bound	Upper Bound
**CIMT Rt. (mm)**	Normal	Overweight	-0.07566	-0.2198	0.0685
	Normal	Obese	-0.19042	-0.3501	-0.0308
	Overweight	Obese	-0.11476	-0.2282	-0.0014
**CIMT Lt. (mm)**	Normal	Overweight	-0.07507	-0.2228	0.0726
	Normal	Obese	-0.18484	-0.3484	-0.0212
	Overweight	Obese	-0.10977	-0.226	0.0064

The mean age of normal, overweight and obese were 40.9 years, 44.93 years and 45.79 years respectively suggesting mean age of overweight and obese were higher than that of normal BMI cases. The CIMT values of both sides were increased in the cases of Kathmandu valley compared to those from outside the Kathmandu valley (Table 4).

**Table 4:** Carotid intima-media thickness (CIMT) as per place of residence.

	Residence	Number	Mean±Standard Deviation	95% Confidence Interval of	
				Lower	Upper
**CIMT Rt. (mm)**	Inside Kathmandu valley	25	0.7368±0.19174	-0.05418	0.12511
	Outside Kathmandu valley	45	0.7013±0.17341		
**CIMT Lt. (mm)**	Inside Kathmandu valley	25	0.7424±0.18121	-0.05026	0.13195
	Outside Kathmandu valley	45	0.7016±0.18402		

The obtained mean differences as per ethnicity showed more increased CIMT values of both sides in Janajati ethnic group as compared to Brahmin/Chhetri, Madhesi and Dalits cases. Likewise Dalits ethnic group had increased values as compared to Brahmin/Chhetric and Madhesi groups while Madhesi ethnic group had increased values as compared to Brahmmin/Chhetric group (Table 5).

**Table 5:** Carotid intima-media thickness (CIMT) as per ethnicity. |

Dependent	(I) Ethnicity	(J) Ethnicity	Mean Difference	Std. Error	95% Confidence Interval I	
Variable			(I-J)		Lower	Upper
**CIMT Rt. (mm)**	Brahmin/Chhetri	Janajati	-0.12671	0.04674	-0.2499	-0.0035
	Brahmin/Chhetri	Madhesi	0G.00716	0.07641	-0.2085	0.1942
	Brahmin/Chhetri	Dalits	-0.08016	0.08272	-0.2982	0.1379
	Janajati	Madhesi	0.11955	0.07996	-0.0912	0.3303
	Janajati	Dalits	0.04655	0.08601	-0.1802	0.2732
	Madhesi	Dalits	-0.073	0.10512	-0.3501	0.2041
**CIMT Lt. (mm)**	Brahmin/Chhetri	Janajati	-0.13452	0.04732	-0.2593	-0.0098
	Brahmin/Chhetri	Madhesi	-0.0077	0.07736	-0.2116	0.1962
	Brahmin/Chhetri	Dalits	-0.0827	0.08375	-0.3035	0.138
	Janajati	Madhesi	0.12682	0.08096	-0.0866	0.3402
	Janajati	Dalits	0.05182	0.08709	-0.1777	0.2813
	Madhesi	Dalits	-0.075	0.10644	-0.3555	0.2055

## DISCUSSION

Nonalcoholic fatty liver disease (NAFLD) is the most common chronic liver disease in the United States and worldwide with increased prevalence currently affecting approximately 30% of adults and 10% of children in the United States in a study done by Wieckowska, et al.^11^ Likewise, in studies done by Browning, et al.^12^ Volzke, et al.^13^ Targher, et al.^14^ Younossi, et al.^15^ the prevalence were around 33%, 29.9%, 36.3%, 24.24% respectively in general population has been mentioned. Thus, prevalence based on different studies suggest it is around 1/3^rd^ among general population which is increasing in recent years. NAFLD represents a wide spectrum of conditions ranging from simple fatty liver which generally follows a benign non-progressive clinical course, to non-alcoholic steatohepatitis (NASH), which is a more serious form of NAFLD that may finally progress to cirrhosis and end-stage liver disease.^1,3,9-12^ The incidence of cardiovascular disease is higher in NAFLD as compared to those without NAFLD and studies have shown it being as a predictor of cardiovascular disease and independent of conventional risk factors.^16-18^

Chouhan, et al.^19^ included 46 NAFLD patients out of which 28 (60.8%) were male and 18 (39.1%) were female, Rasool, et al.^20^ had 200 NAFLD study population comprising of 86 (43%) male and 114 (57%) female. Likewise, Kejriwal, et al.^21^ had 100 NAFLD study population out of which 59 (59%) were male and 41 (41%) were female and Zayed, et al.^22^ took 70 NAFLD patients in his study which included 33 (47.1%) male and 37(52.8%) female. In our study, we took 70 NAFLD cases comprising of 33 (48.5%) male and 37 (51.4%) female. Thus our study sample was comparable with these studies.

In a study conducted by Kejriwal, et al.^21^ patients of age group 30 to 60 years were studied as we studied in same age groups where we had maximum frequency of NAFLD in the age group of 30-35 years as 22.9% vs 25% showing our results were comparable with their study.

In a study by Rasool, et al.^20^ Grade I fatty liver was seen in 36% patients while Grade II fatty liver was found in 39% and Grade III fatty liver in 25% patients. Chouhan, et al.^18^ reported that 54.34% patients had grade I fatty liver, 41.30% had grade II fatty liver and 4.34% had grade III fatty liver. Study done by Kejriwal, et al.^21^ reported Grade I fatty liver in 52%, Grade II in 42% and Grade III in 6%. In our study, we had 35.7% of Grade I fatty liver, and 64.3% of Grade II fatty liver and none of our study population had Grade III fatty liver case which could be attributed to under-diagnosis. In the study by Chouhan, et al.^19^ and Kejriwal, et al.^21^ they had Grade III fatty liver in NAFLD patients in their study, but the numbers were too low.

In a study by Guleria, et al.^23^ mean CIMT was of 0.70±0.11mm while mean CIMT of right and left side were 0.69±0.12mm and 0.72±0.12mm respectively which were significantly increased as compared to controls. Study done by Rasool, et al.^20^ observed mean CIMT of right and left side as 0.84566mm and 0.80202mm respectively. Mean CIMT was 0.69mm and 0.71mm in Grade I fatty liver in left and right side respectively. Similarly in Grade II fatty liver mean CIMT of 0.84mm and 0.80mm in right and left side respectively. There was increased CIMT in cases than controls progressively increasing with higher grades of fatty liver which was statistically significant. Observational study by Kejriwal, et al.^21^ showed CIMT of 0.0629 ±0.00860mm, of 0.0792 ±0.01004mm and of 0.0860 ±0.00490mm in Grade I, Grade II and Grade III fatty liver respectively on right side. Similarly on left side CIMT measured were 0.0654±0.01012mm, of 0.0799 ±0.00893mm and 0.0900 ±0.00580mm in Grade I, Grade II and Grade III fatty liver respectively. In our study, the mean CIMT of 0.7151±0.1805mm was noted along with mean CIMT of 0.7140±0.1796mm and 0.7161±0.1828mm on right and left side respectively. The mean CIMT was 0.5720±0.1275mm and 0.7929±0.1546mm in Grade I and II NAFLD cases respectively on right side whereas mean CIMT of 0.5676±0.1259mm and 0.7987±0.1557mm was noted in Grade I and II NAFLD cases respectively on left side. There was increase in CIMT values of both sides in Grade II NAFLD as compared to Grade I NAFLD. Thus the overall mean CIMT, mean CIMT of both sides and CIMT in different grades of fatty liver were comparable with above studies' results although our study was only descriptive cross sectional study. Results from the studies by Zayed, et al.^22^ and Chouhan, et al.^19^ also support our study results. The studies by Riaz, et al.,^24^ Targher et al.,^25^ Sookoian and Pirola^26^ showed positive association between NAFLD and raised CIMT which was statistically significant validating our study results.

Riaz, et al.^24^ stratified CIMT with respect to BMI in to groups BMI ≤30kg/m^2^ and BMI >30kg/m^2^ where 55.96% and 44.04% of NAFLD cases had BMI ≤30kg/m^2^ and BMI >30kg/m^2^ respectively. Their results showed statistically significant association between NAFLD and raised CIMT in BMI >30kg/ m^2^. In our study, 72.86% NAFLD cases had BMI ≤30kg/m^2^ while 19 (27.14%) had BMI >30kg/m^2^. Our results showed increase in CIMT in obese (BMI ≥30kg/m^2^) as compared to overweight (BMI <30kg/m^2^ and ≥25kg/m^2^) and normal (BMI <25kg/m^2^) cases on both sides. Thus our results were supported by the above studies.

There were increased CIMT values in Janajati ethnic groups as compared to Brahmin/Chhetri groups in our study. There were also increased CIMT values in cases from the Kathmandu valley as compared to those outside the Kathmandu valley. No similar studies/literatures which included these variables are available to compare our results. The probable cause of the increase in the values could be due to overweight and obese leading to fatty liver in these groups as compared to other ethnic groups as a result of their life style including their eating habits. More studies and researches (including analytical study) taking large sample size and controls to measure their associations are required to support these results and to draw conclusions.

Till date, the liver biopsy remains the only reliable way to diagnose NASH and establish the presence of fibrosis. Current noninvasive clinically available tests lack accuracy and reliability. So, in the scenario of rapid increase in the prevalence of NAFLD, the significant research effort in developing novel therapies for NASH patients along with noninvasive, simple, reproducible and reliable biomarkers are greatly needed which will not only help in the diagnosis of NASH, but also be useful for assessment of treatment response and prognosis. So, this should remain a research priority in the NAFLD field to prevent NAFLD related morbidity and mortality.

Our study has some limitations. The sample size was small. Our study was descriptive cross sectional study with no controls enrolled. NAFLD which is one of the important causes of increased CIMT was only studied in our study while other parameters like DM, HTN, obesity, hypercholesterolemia, chronic liver disease, smoking etc. which have profound effect on CIMT were excluded just based on clinical history. The detailed clinical examinations and relevant laboratory investigations were not carried out. The diagnosis of NAFLD was exclusively based on ultrasound findings although liver biopsy is the gold standard to diagnose NAFLD which was not carried out in our study.

## CONCLUSIONS

Our study showed an increase in CIMT in NAFLD cases with differences in CIMT measurements in different grades of fatty liver, with BMI values, ethnicity and place of residence. Our study showed increased CIMT values with higher grades of fatty liver, higher values of BMI, in Janajati ethnic groups and cases from Kathmandu valley. There is an increase in CIMT in cases of carotid atherosclerosis. Thus, CIMT measurement in NAFLD patients is one of the valuable indicators for assessing carotid atherosclerosis which is an important risk factor for cardiovascular diseases. So, carotid ultrasound is easily available, a cost effective and non-invasive tool for evaluating CIMT to assess carotid atherosclerosis. Thus, an early intervention can be started if there is an increase in CIMT to minimize the possible complications of atherosclerosis in the form of various cardiovascular diseases or events.
